# Vitamin C: Rationale for Its Use in Sepsis-Induced Acute Respiratory Distress Syndrome (ARDS)

**DOI:** 10.3390/antiox13010095

**Published:** 2024-01-12

**Authors:** Alpha A. Fowler

**Affiliations:** Division of Pulmonary Disease and Critical Care Medicine, Department of Internal Medicine, School of Medicine, Virginia Commonwealth University, Richmond, VA 23219, USA; alpha.fowler@vcuhealth.org

**Keywords:** sepsis, ARDS, vitamin C, neutrophil extracellular traps (NETS), NETosis, reactive oxygen species (ROS), lung vascular injury, cell-free hemoglobin (CFH), sodium vitamin C transporters SVCT 1 and 2

## Abstract

Acute respiratory distress syndrome (ARDS) is a life-threatening event that occurs in patients suffering from bacterial, fungal, or viral sepsis. Research performed over the last five decades showed that ARDS is a consequence of severe unrestrained systemic inflammation, which leads to injury of the lung’s microvasculature and alveolar epithelium. ARDS leads to acute hypoxic/hypercapnic respiratory failure and death in a significant number of patients hospitalized in intensive care units worldwide. Basic and clinical research performed during the time since ARDS was first described has been unable to construct a pharmacological agent that will combat the inflammatory fire leading to ARDS. In-depth studies of the molecular pharmacology of vitamin C indicate that it can serve as a potent anti-inflammatory agent capable of attenuating the pathobiological events that lead to acute injury of the lungs and other body organs. This analysis of vitamin C’s role in the treatment of ARDS includes a focused systematic review of the literature relevant to the molecular physiology of vitamin C and to the past performance of clinical trials using the agent.

## 1. Introduction

Acute respiratory distress syndrome (ARDS) is an acute inflammatory injury of the lung’s microvasculature and alveolar epithelium. Increased lung capillary permeability produces loss of the lung’s microvascular barrier function leading to hypoxia and non-cardiogenic pulmonary edema. Loss of barrier function leads to acute hypoxic/hypercapnic respiratory failure with frequent mortalities of 35% to 40% [1,2]. ARDS is typically acute in onset; however, delayed development (up to 48 h) often occurs [3]. Pneumonia and non-pulmonary sepsis (e.g., abdominal, skin, and catheter related) are leading causes of ARDS development [4]. Toxic inhalation [5], transfusion-associated lung injury [6], near drowning [7], and lung injury induced by drugs [8] are less frequent causes of ARDS. ARDS was first reported over 50 years ago by Ashbaugh and colleagues who described acute respiratory distress syndrome in 12 patients characterized by severe dyspnea, tachypnea, cyanosis unresponsive to oxygen therapy, and importantly, loss of lung compliance. They noted diffuse airspace disease radiographically with an associated high mortality rate [9]. Since the initial report, multiple observational studies have helped clarify the epidemiology of ARDS [10,11]. However, despite significant basic research along with an improved understanding of the pathophysiology of ARDS [12,13], no definitive pharmacologic therapy capable of attenuating lung injury has arisen. Vitamin C, also known as L-ascorbic acid, is a water-soluble vitamin that acts physiologically as a potent antioxidant that limits oxidative stress. In addition to its biosynthetic (i.e., collagen, L-carnitine, and neurotransmitter synthesis) and antioxidant functions, vitamin C plays a significant role in immune function. In this review, an in-depth review of vitamin C’s molecular pharmacology, along with data to provide an enhanced understanding of the rationale for use of intravenous vitamin C as a therapy for sepsis-induced ARDS, is summarized.

**1.**  **Loss of Lung Barrier Function: Capillary Endothelial and Alveolar Epithelial Injury Result from Uncontrolled Inflammation**

**Figure 1:**
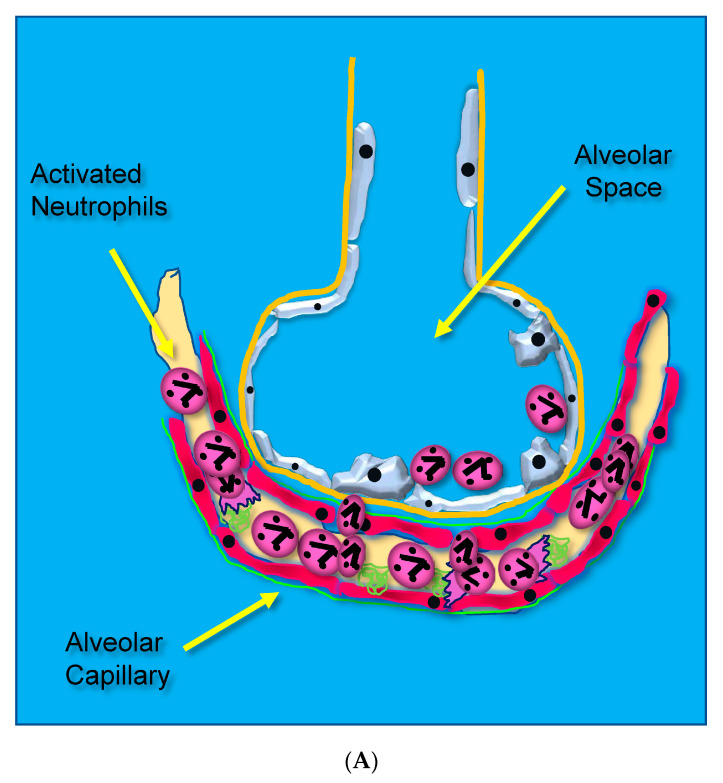

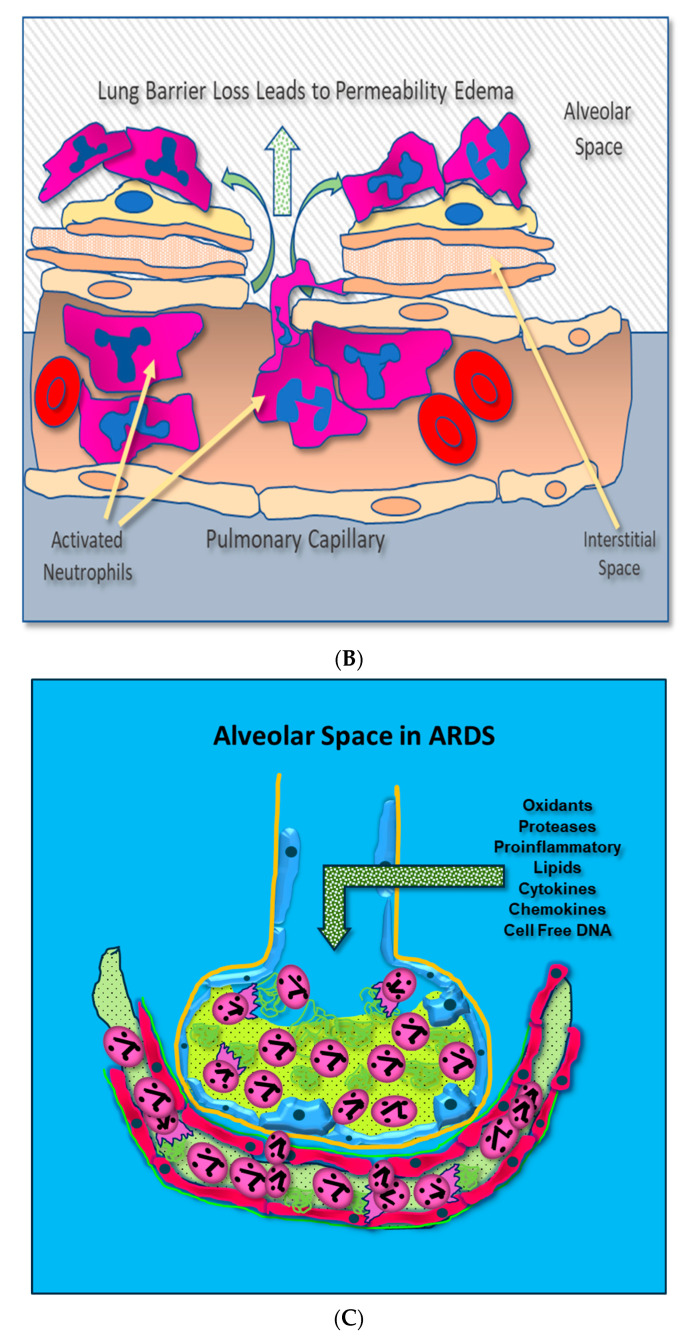
(**A**) Activated neutrophils adhere to the surfaces of pulmonary capillaries following the onset of sepsis. (**B**) Activated neutrophils damage pulmonary capillary surfaces, which lead to the disruption of the alveolar capillary membrane and loss of lung barrier function. (**C**) Activated adherent neutrophils transmigrate into the alveolar space. Oxidants, proteases, proinflammatory lipids, cell-free DNA, and potent cytokines and chemokines damage constituents of the alveolar space, further promoting inflammatory injury.

Acute lung injury leading to ARDS occurs through unrestrained dysregulated inflammation that injures capillary endothelial and alveolar epithelial cells, which damages the barrier between the lung’s capillary vasculature and airspaces. Following the onset of sepsis, circulating microbes and microbial toxins (e.g., lipopolysaccharide, LPS) bind to toll-like receptors (TLRs) on endothelial surfaces promoting alarm-type innate immune responses [14]. Activated endothelial TLRs upregulate endothelial cell cytokine and chemokine secretion driven by transcription factor nuclear factor kappa B (NF-κB). Upregulated adhesion molecule expression [(i.e., selectins, vascular cell adhesion molecule, (VCAM), and intercellular adhesion molecule, (ICAM-1)] [15] causes leukocytes to adhere to the capillary endothelium (Figure 1A) [16].

**Figure 2:**
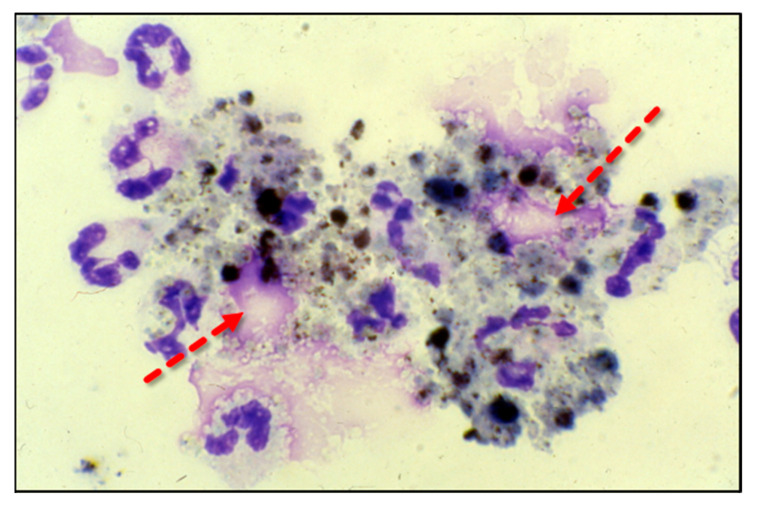
ARDS bronchoalveolar lavage cytology showing neutrophils extruding extracellular traps (arrows).

Adherent neutrophils produce damaging reactive oxygen species (ROS), secrete proteases, and extrude genomic DNA with intact activated proteases that form neutrophil extracellular traps (NETs) [17,18,19,20]. Endothelial damage disrupts the alveolar–capillary membrane barrier (Figure 1B), leading to neutrophil trans-endothelial migration into the alveolar space. Activated neutrophils and plasma from capillary circulation flow into the previously dry lung airspaces (Figure 1C). Lung water subsequently increases as the alveolar space becomes a proinflammatory environment localizing ROS, inflammatory lipids, cytokines, chemokines, and NETs containing elastase, myeloperoxidase, cathepsin G, and metalloproteases in the alveolar space. (Figure 1C) [21,22,23]. Using bronchoalveolar lavage (BAL) early in the course of ARDS reveals the toxic inflammatory environment present in the alveolar space (Figure 2). Arrows indicate NETs in the alveolar space (AA Fowler, unpublished image). The resulting inflammatory environment in the alveolar space damages type 1 and 2 alveolar epithelial cells [24], which then damages the molecular water channels necessary for the clearance of alveolar fluid.

Ware and Matthay performed alveolar fluid clearance (AFC) studies in patients with ARDS [25]. In these studies, a submaximal or impaired alveolar fluid clearance in ARDS patients predicted a 20% and 62% mortality, respectively. An impaired or submaximal AFC predicted a significantly higher number of days of assisted mechanical ventilation. As a result, ARDS accounts for 33% of all ICU patient days and 24% of hospital charges among ICU patients, making ARDS one of the costliest ICU diagnoses. Boucher et al. systematically searched the literature for articles relevant to ARDS costs [26]. In this report, costs were surveyed in 49,483 patients with ARDS. Costs per ARDS patient ranged between USD 54,490 and USD 450,888 [26]. The least expensive costs occurred in a publicly funded healthcare system in Finland, with the most expensive costs in a private hospital system in the United States. Understanding healthcare and human costs related to ARDS is vital. Biomedical research into cost-effective interventions that alter morbidity and mortality is crucial for a syndrome that produces high death rates worldwide. From a pathophysiological perspective, a multi-functional agent capable of reducing the *“inflammatory fire”* in the lungs of ARDS patients is needed. Preclinical and human trial research into the rationale for use of vitamin C as an adjunctive therapy for ARDS is described below.

## 2. Cellular Transport of “Reduced” Vitamin C

Vitamin C is transported into cells via two forms of sodium-dependent vitamin C transporters (SVCT1 and SVCT2). Both transporters mediate a high affinity for sodium and the energy-dependent transport of vitamin C into cells [27]. SVCT1, expressed predominantly in epithelial tissues (e.g., intestine, liver, kidney, skin), contributes to the supply and maintenance of whole-body ascorbic acid levels. SVCT2 is more widely distributed (i.e., endothelium, brain, lung, liver, skin, spleen, muscle, adrenal gland). SVCT2 is the major transporter present in lung endothelium and epithelium and mediates vitamin C transport into the alveolar space [28,29]. SVCT2 establishes steep concentration gradients of vitamin C across plasma cell membranes as well as transport from the cytosol to the mitochondrial matrices [30]. Covarrubias-Pinto et al. showed that extracellular ascorbic acid increases SVCT2 expression at the plasma membrane by accelerating its trafficking from intracellular secretory compartments to cell membranes. Enhanced cellular SVCT2 expression then increases vitamin C transport across cell membranes, producing increased intracellular vitamin C concentrations in immune cells such as neutrophils, macrophages, and lymphocytes [31,32,33,34]. Metnitz et al. reported that plasma vitamin C levels are low in critically ill patients with polymicrobial sepsis that leads to ARDS [35]. Covarrubias-Pinto’s work suggests that cellular SVCT2 expression is low in patients with sepsis, a state characterized by high level oxidative stress. Thus, in order for SVCT2 expression to be increased, vitamin C must be infused intravenously to increase peri-cellular concentrations [36]. The protection of capillary blood flow and arteriolar responsiveness by vitamin C may be mediated by the inhibition of oxidative stress, the modulation of intracellular signaling pathways, and the maintenance of homeostatic levels of nitric oxide. Vitamin C scavenges reactive oxygen species (see below). NADPH oxidase, which synthesizes superoxide anion in microvascular endothelial cells, is inhibited/scavenged by vitamin C [37].

## 3. Neutrophil Extracellular Traps (NETs): Their Role in Lung Injury and the Impact of Vitamin C Infusion

**Figure 3:**
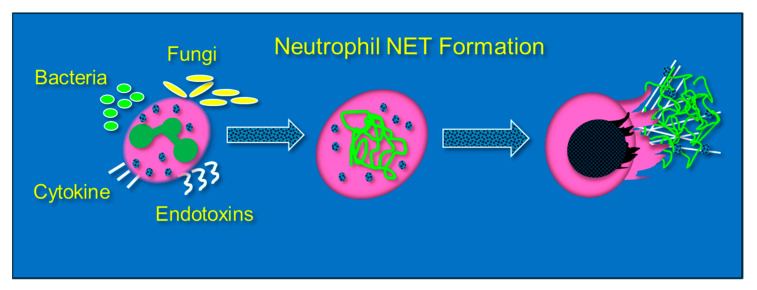
Neutrophils extracellularly extrude genomic contents and active enzymes following exposure to bacteria, endotoxins, fungi, and certain cytokines and chemokines.

Neutrophils are phagocytic cells that defend against pathogens by ingestion and then killing via oxidant or protease-dependent mechanisms [38]. The formation of NETs, or NETosis, is initiated by a sudden rise in intracellular calcium that unravels nuclear chromatin with subsequent cellular expulsion, complete with cytoplasmic granular proteins, into the extracellular space. NETosis occurs when neutrophils encounter an endotoxin from bacteria, viruses, fungi, or certain proinflammatory peptides (e.g., cytokines and chemokines) (Figure 3). NET composition is significantly proinflammatory due to the presence of granule-derived proteases (e.g., elastase and serine proteases), histones, and cell-free DNA, which forms the structural support for NETs and which itself is proinflammatory [39,40]. Although NETosis is a response to infection, the presence of NETs promotes the damage of healthy tissue by direct cellular injury [41]. Lefrancais et al. showed that abundant NET formation was present in murine models of acute lung injury [42]. Although NET formation promotes pathogen clearance, NET formation, particularly in the lungs, intensifies inflammation and tissue injury [43]. Gupta et al. found that cytokine-activated endothelial cells, characteristic of sepsis, promoted NETosis, which subsequently led to endothelial cell death [44]. BAL fluid from septic humans with ARDS reveals the presence of NETs, which also reveals that transmigrating neutrophils undergo NETosis [45]. Importantly, the presence of NETs in the alveolar space leads alveolar macrophages to polarize to M1 phenotypes, which then further promotes lung injury [46]. LPS that is produced during intraperitoneal sepsis is a key factor in triggering NETosis and leads to acute lung and non-pulmonary organ injury [47,48]. Mohammed and colleagues showed that feces-induced peritonitis promoted NET formation in the lungs of wild-type mice. In Mohammed’s studies, lung NET formation was significantly inhibited by the infusion of high dosage parenteral vitamin C [49]. Mohammed performed further studies that examined NETosis induced in vitro through phorbol myristate acetate exposure and showed significant NET attenuation by parenteral vitamin C [49]. Systemic NETosis in the setting of sepsis and fatal organ injury has been assessed through analysis of plasma cell-free DNA (cfDNA) [50,51]. Higher concentrations of circulating NETs are associated with worsened clinical outcomes and organ failure in patients with sepsis [52,53]. The inhibition of one component of NETs (neutrophil elastase) was unsuccessful in altering the mortality of ARDS patients [54].

**Figure 4:**
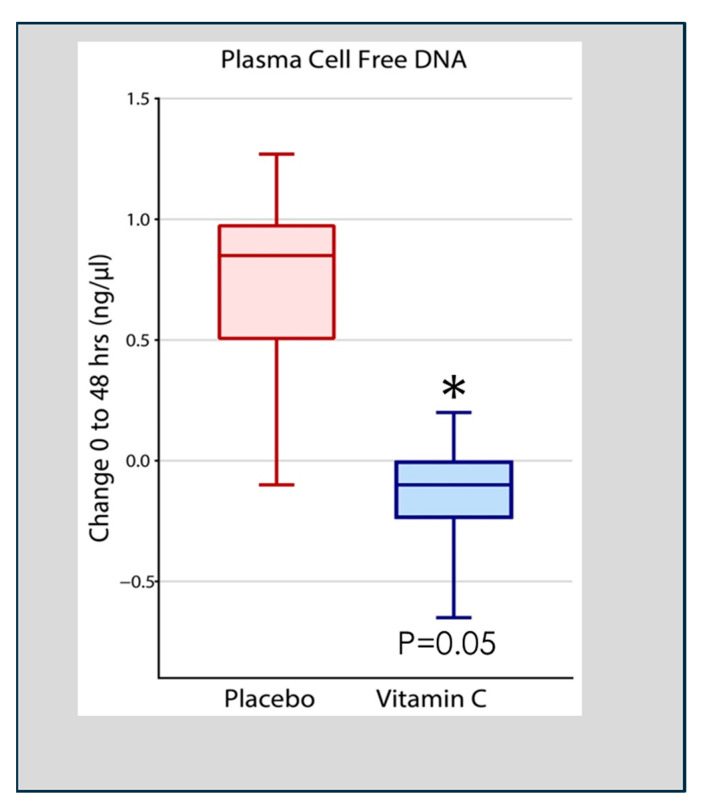
Vitamin C infusion significantly reduced plasma cell-free DNA concentrations following the onset of sepsis-induced ARDS. Asterisk indicates significant reduction.

Qiao et al. analyzed cell-free DNA in plasma obtained at 48 h following the onset of ARDS in 167 patients enrolled in the CITRIS-ALI trial in which patients with sepsis-induced ARDS were treated with high-dose intravenous vitamin C. Qiao found that patients treated with intravenous vitamin C, compared to placebo-treated patients, exhibited significant reductions in plasma cell-free DNA (Figure 4) [55]. Previous pre-clinical studies in vitamin C-deficient L gulonolactone oxidase (Gulo) knockout mice showed enhanced NETosis in the lungs of septic animals and increased circulating cell-free DNA [49]. These results suggest that the high plasma levels of vitamin C achieved by intravenous infusion played a significant role in the degradation of circulating NETs in the study by Qiao et al. [55]. Although the process of NETs generation and their associated pathophysiology is known, knowledge of their degradation and the attenuation of the extent of NETs-injured tissues is scarce [56]. Haider et al. suggested that NETs are cleaved by secreted extracellular DNases followed by intracellular degradation in macrophages that digest phagocytosed fragments of extracellular DNA [57]. This process is facilitated by the extracellular digestion of large fragments of NETs by DNase I secreted by macrophages [58]. As DNA is the main component of NETs, DNases have emerged as fundamental enzymes that breakdown NETs in vivo [59]. Thus, as has been reported, defects in the dismantling or degradation of NETs, as is apparent in patients with sepsis and ARDS, is likely instrumental in promoting organ injury and mortality. The CITRIS-ALI trial found significant reductions in mortality and organ injury in sepsis-induced ARDS [60,61] with significant reductions in plasma cell-free DNA (i.e., NETs) [55], as was also found in vitamin C-treated septic animal studies [49]. These studies suggest that vitamin C is intimately involved in dismantling circulating NETs, which likely played a role in the outcome of patients with sepsis-induced ARDS in the CITRIS-ALI trial.

## 4. Pattern Recognition Receptors Drive Cytokine/Chemokine Expression: The Role of Vitamin C in Attenuating Proinflammatory NF-κB Expression

Pattern recognition receptors (PRRs) recognize pathogen-associated molecular patterns (PAMPs) and endogenous danger-associated molecular patterns (DAMPs). DAMPs (e.g., High mobility group box 1, HMGB1) are host nuclear or cytoplasmic non-microbial molecules that, when released from the cell following tissue injury, serve as potent activators of the immune system, initiating and perpetuating a non-infectious inflammatory response [62]. Once engaged, PRRs fuel the expression of NF-κB driven cytokine and chemokine expression (e.g., tumor necrosis factor-α (TNFα), interleukin-1β (IL-1β), IL-8, IL-6) [63], which promotes autophagy and apoptosis and induces the expression of TLRs, which are intimately involved with development of ARDS. Meduri et al. and Hyers et al. analyzed BAL specimens from patients with ARDS and showed that levels of TNF-α, IL-1β, and IL-8 correlated with BAL fluid indices of endothelial permeability (i.e., loss of lung barrier function) [64,65]. In Meduri’s studies, over time, BAL to plasma concentration ratios of TNF-α, 1L-1β, and IL-6 remained elevated in non-survivors and decreased in survivors [64].

**Figure 5:**
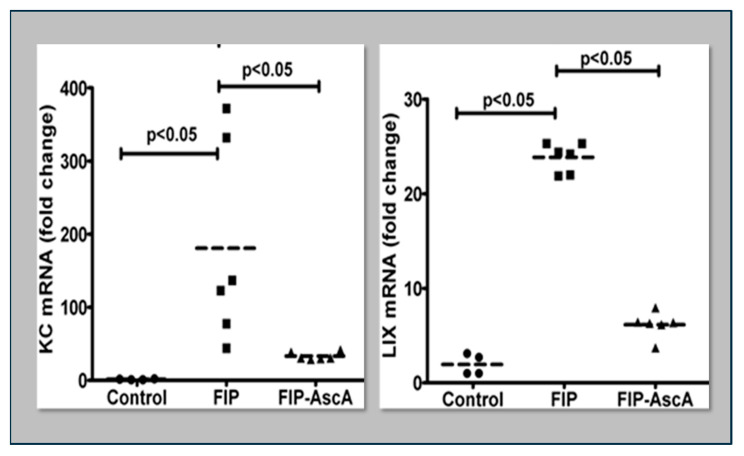
Parenteral vitamin C infusion in mice with peritoneal sepsis significantly reduced lung mRNA content of the proinflammatory chemokines KC and LIX. FIP = Feces-Induced Peritonitis, AscA = Ascorbic Acid.

**Figure 6:**
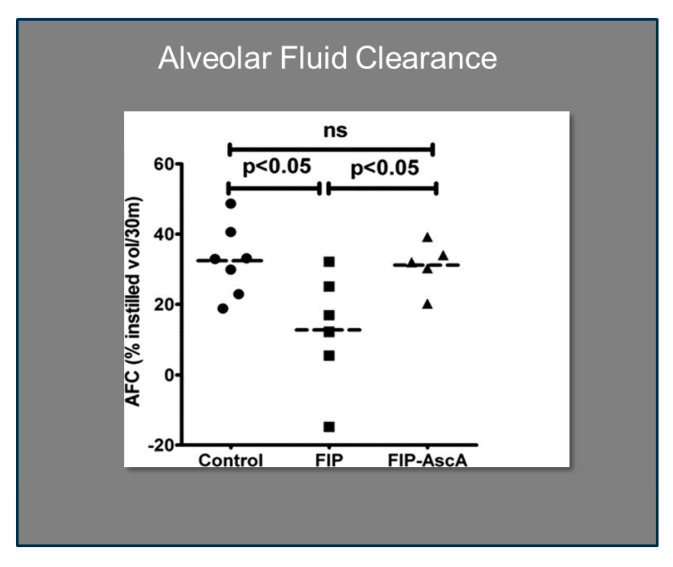
Vitamin C significantly improves alveolar fluid clearance in septic mice. FIP = Feces-induced Peritonitis, AscA = Ascorbic Acid.

Williams et al. found that chemokines CCL2 and CCL7 are elevated in BAL fluid from patients with ARDS and that these chemokines promote chemotactic activity in ARDS BAL fluid by synergizing with the chemokine CXCL8 to promote neutrophil migration into the alveolar space [66]. Thus, attenuating the acute inflammatory response driven by cytokine and chemokine expression in ARDS lungs is a critical element in treatment. Fisher et al. created a model of sepsis-induced acute lung injury by injecting fecal stem solution into the peritoneum of wild-type mice [67]. In Fisher’s model, feces-induced peritonitis induced acute lung injury with significant increases in BAL protein and lung water. The BAL fluid cellular analysis in Fisher’s studies revealed intense neutrophil migration into the alveolar space with significant increases in lung myeloperoxidase mRNA, documenting the extent of neutrophil sequestration in septic lungs. When animals were treated with high dosage parenteral vitamin C, Fisher found significant reductions in lung chemokine (KC and LIX) expression (Figure 5), in lung HMGB1 expression, and importantly, significant reductions in lung water accumulation and BAL protein content. The vitamin C infusion in Fisher’s studies of feces-induced lung injury augmented the epithelial ion channel/transporter expression (i.e., Na^+^/K^+^ATPase, aquaporin 5), and this expression was associated with significant increases in alveolar fluid clearance in septic mice (Figure 6) [67]. In further studies using the feces-induced peritonitis model of acute lung injury, Fisher and colleagues showed that vitamin C infusion significantly attenuated NF-κB activation, which occurred following the onset of sepsis-induced acute lung injury [68].

## 5. Reactive Oxygen Species (ROS) in ARDS: Is There a Role for Vitamin C?

There is significant evidence that supports a role for ROS in the pathogenesis of ARDS. Over 40 years ago, Tate and colleagues showed that infusion of the oxidant-generating enzyme xanthine oxidase into an isolated perfused rabbit lung induced increased alveolar capillary membrane permeability as evidenced by the increased BAL protein content [69]. In later whole animal studies, Brigham and Meyrick showed that capillary endothelial injury in the lungs was dependent upon activated neutrophil sequestration [70]. These early data support the concept that ROS, generated by chemical means (i.e., xanthine oxidase) or by activated neutrophils generating ROS, play a key role in creating acute lung injury. Thus, neutrophils, when activated by infectious stimuli, sequester in the lungs and promote endothelial injury, supporting the concept that the close apposition of activated neutrophils with pulmonary capillary endothelium is critical for the generation of lung injury. Park et al. imaged acute lung injury in vivo as it evolved by employing a real-time intravital lung microscopic imaging system, which documented prolonged neutrophil entrapment in lung capillaries during sepsis-induced acute lung injury in mice [71]. Adherent activated neutrophils generate significant quantities of superoxide, hydrogen peroxide, and reactive nitrogen products. Suzuki et al. showed that dismuting ROS with superoxide dismutase attenuates sepsis-induced acute lung injury [72]. Many other studies employing animal models, which have employed antioxidants, have been conducted over the years, demonstrating the efficacy of antioxidants in attenuating ROS-induced lung injury [73,74,75,76].

**Figure 7:**
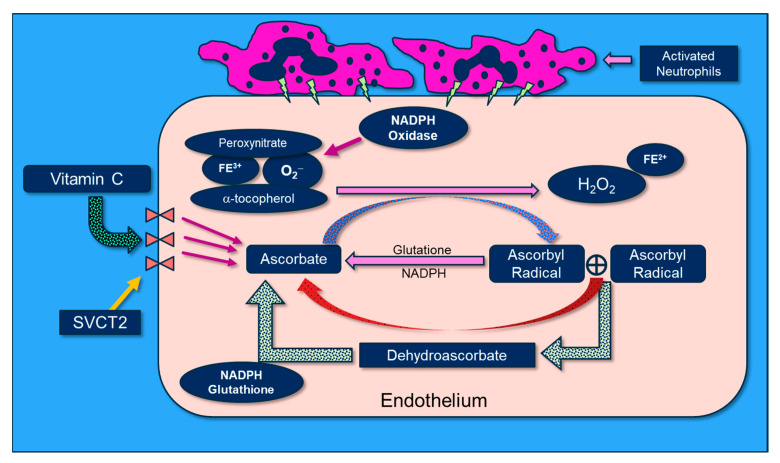
The molecular physiology of vitamin C’s attenuation of reactive oxygen species.

Vitamin C scavenges ROS such as superoxide (O_2_^−^) and peroxynitrite (ONOO^−^) in plasma and cells, thus preventing damage to proteins, lipids, and DNA. By donating an electron, vitamin C is able to “repair” oxidized antioxidants (e.g., glutathione and α-tocopherol). Following vitamin C’s electron donation, unstable ascorbyl radicals (AR) are formed. Subsequently, two AR radicals rapidly “dismute” to reform ascorbate (vitamin C) and dehydroascorbate. Dehydroascorbate is reduced by interaction with glutathione and NADH-dependent dehydrogenases (Figure 7). Vitamin C’s biochemical actions inhibit NADPH oxidase, and it potently scavenges O2^−^ as well as OONO^-^, thus preventing/attenuating cellular injury. Further, vitamin C reduces oxidized tetrahydrobiopterin (BH_2_) to BH_4,_ a critical cofactor of endothelial nitric oxide synthase (eNOS), which increases bioavailable nitric oxide that helps restore microvascular perfusion [77,78,79]. Multiple lines of research support a role for vitamin C in attenuating oxidative-induced lung injury. Patel et al. and Mohamed et al. demonstrated that parenteral administration of vitamin C attenuated hyperoxia and sepsis-induced acute lung injury [80,81]. Dwenger et al. found that vitamin C infusion significantly attenuated endotoxin-induced lung injury in sheep [82]. The CITRIS-ALI trial was a randomized, double-blind, placebo-controlled, and multicenter trial that included 167 patients with sepsis-induced ARDS who were admitted to seven medical intensive care units (ICUs) in the United States. Patients were randomly assigned to receive either IV vitamin C at 50 mg/kg (*n* = 84) or a placebo (*n* = 83) every 6 h for 96 h [60]. Although no differences were found in the biomarker analysis of the C-reactive protein or thrombomodulin, reanalysis of the sequential organ failure assessment scores found that vitamin C significantly lowered sepsis-induced organ injury [61]. All-cause mortality in the CITRIS-ALI trial was significantly reduced in patients infused with vitamin C [60]. Zhang and colleagues studied 54 patients with COVID-19-induced ARDS. Patients were randomly assigned in a 1:1 ratio to receive either IV infusion of 12 g of vitamin C or a placebo every 12 h for 7 days. They found no difference in ventilation-free days; however, the vitamin C group exhibited significant improvements in the ratio of PaO_2_ to the fraction of inspired oxygen at day 7 in the vitamin C arm (229 vs. 151 mm Hg, 95% CI 33 to 122; *p* = 0.01), as well as a trend in the reduction in the 28-day mortality in a subgroup of patients with SOFA scores of ≥3 receiving vitamin C (*p* = 0.06) [83].

## 6. Cell-Free Hemoglobin-Induced Vascular Injury: A Role for Vitamin C

Sepsis leading to acute lung injury promotes red cell membrane fragility, which induces hemolysis, resulting in the release of cell-free hemoglobin (CFH) in a high percentage of septic patients [84]. Typically, CFH rapidly binds to haptoglobin and hemopexin; however, large amounts of CFH, such as are present in septic patients, represent a potent stimulus driving neutrophil activation and migration [85,86]. Adamzik et al. reported that elevated cell-free hemoglobin concentrations from patients with severe sepsis were associated with an increased 30-day mortality [87]. When released from erythrocytes into pericellular environments, CFH is a potent pro-oxidant that can react with key proteins, lipids, and DNA [88]. CFH promotes increased microvascular permeability. Shaver et al. reported that CFH in the airspace is a driver of lung epithelial injury in human and experimental ARDS [89]. Meegan et al. found that infusion of CFH into septic wild-type mice increased endothelial injury, vascular permeability, systemic inflammation, and organ injury [90]. Shaver et al. found that cell-free hemoglobin increased pulmonary edema and vascular permeability in ex vivo isolated perfused human lungs [91]. Intracellular ascorbate promotes enhanced endothelial barrier function by impacting the tubulin cytoskeleton [92]. Importantly, Kuck et al. found that as CFH concentrations were increased, intracellular ascorbate was depleted [93]. Supplementing increased concentrations of vitamin C in Kuck’s studies reversed the permeability of endothelial monolayers induced by CFH [93]. In a recent abstract publication, Shaver et al. analyzed CFH concentrations in the plasma of ARDS patients in the CITRIS-ALI trial and showed that the mortality benefit of vitamin C was highest in the patients exposed to the highest FiO_2_. In these studies, the mortality benefit of vitamin C was present only in the patients with elevated plasma CFH [94]. Although there are implications of the molecular interactions of iron with vitamin C, these interactions have not yet been fully characterized. Currently, clinical evidence concerning the effects of the molecular interactions of iron and vitamin C are obtained from the use of ascorbic acid in iron overload states [95].

## 7. Is There a Role for Parenteral Vitamin C in ARDS?

Current evidence shows that administration of parenteral vitamin C in critically ill patients is safe [96]. Fang et al. performed a meta-analysis of vitamin C in patients with sepsis and septic shock and found that the use of vitamin C (compared with placebo) led to reduced ICU mortality and reductions in vasopressor dosage in patients with septic shock [97]. A more recent meta-analysis by Liang et al. on sepsis or septic shock significantly improved the delta SOFA scores and reduced the duration of vasopressor use but was not associated with reduced short-term mortality [98]. Study outcomes for sepsis alone report differing findings with using vitamin C to improve clinical outcomes in critically ill patients [99,100,101]. However, septic processes that lead to lung injury may represent new thinking concerning a potential role for parenteral vitamin C in the treatment of developing or developed ARDS. The limitations of the previous sepsis trials may have resulted from the dosing of the vitamin C utilized, the time after the onset of organ injury, and possibly the preparation of the vitamin C utilized. As reported above, the CITRIS-ALI trial reported that IV infusion of vitamin C at 50 mg/kg every 6 h for 96 h significantly reduced ARDS mortality. Bharara et al. and Fowler et al. reported that infusion of high-dose IV vitamin C was effective in treating recurrent sepsis-induced ARDS as well as rhinovirus/enterovirus-induced ARDS [102,103]. Parenteral vitamin C may have a role in ARDS resulting from SARS-CoV-2-induced ARDS. Gao et al. examined 76 patients with COVID-19-induced ARDS who were infused with high-dose vitamin C and found a significantly reduced mortality rate and biomarkers of inflammation [104]. Based on extensive research regarding the molecular pharmacology of vitamin C and the mechanisms whereby vitamin C alters the proinflammatory states that lead to ARDS, a treatment regimen of intravenous vitamin C may help attenuate the lung injury of ARDS. The intravenous dosing of vitamin C to produce the 800 to 1500 millimolar concentrations for a minimum of 96 h, as was accomplished in the CITRIS-ALI trial, likely produced vitamin C’s anti-inflammatory effects and the outcomes found in the trial. Future trials that focus more specifically on ARDS or the development of ARDS are justified based on the compelling findings from preclinical and observational research.

## Data Availability

Not applicable.
